# Repeating Numbers Reduces Results: Violations of the Identity Axiom in Mental Arithmetic

**DOI:** 10.3389/fpsyg.2018.02453

**Published:** 2018-12-05

**Authors:** Martin H. Fischer, Samuel Shaki

**Affiliations:** ^1^Division of Cognitive Science, University of Potsdam, Potsdam, Germany; ^2^Department of Behavioral Sciences, Ariel University, Ariel, Israel

**Keywords:** AHAB, cognitive bias, mental arithmetic, numerical cognition, operational momentum, SNARC, tie problems

## Abstract

Even simple mental arithmetic is fraught with cognitive biases. For example, adding repeated numbers (so-called tie problems, e.g., 2 + 2) not only has a speed and accuracy advantage over adding different numbers (e.g., 1 + 3) but may also lead to under-representation of the result relative to a standard value (Charras et al., 2012, 2014). Does the tie advantage merely reflect easier encoding or retrieval compared to non-ties, or also a distorted result representation? To answer this question, 47 healthy adults performed two tasks, both of which indicated under-representation of tie results: In a result-to-position pointing task (Experiment 1) we measured the spatial mapping of numbers and found a left-bias for tie compared to non-tie problems. In a result-to-line-length production task (Experiment 2) we measured the underlying magnitude representation directly and obtained shorter lines for tie- compared to non-tie problems. These observations suggest that the processing benefit of tie problems comes at the cost of representational reduction of result meaning. This conclusion is discussed in the context of a recent model of arithmetic heuristics and biases.

## Repeating Numbers Reduces Results: Evidence From Pointing and Production

Human cognition is thought to be a pinnacle of evolution, yet on closer inspection it is fraught with biases (e.g., Tversky and Kahneman, 1974; Evans, 1989; Kahneman, 2012). This is true even for domains of supposedly rational reasoning such as logic or mathematics. In logical reasoning we tend to deny the antecedent or affirm the consequent (Wason, 1966). In mathematics, we fail to correctly solve even simple additions under certain circumstances: Once there is uncertainty in the expression of arithmetic results, we violate the commutativity axiom (a + b = b + a: Shaki et al., 2015) or accept larger than the correct outcomes for simple additions (recent reviews in Fischer and Shaki, 2018; Pinheiro-Chagas et al., 2018). Given the profound importance of rational reasoning for everyday life we need to better understand the sources of such surprising errors. Here we focus on a novel violation of the identity axiom for sums s (s1 = s2) where s1 = a + a and s2 = b + c and a + a = b + c.

The starting point of the present investigation was a series of speeded classification experiments performed by Charras et al. (2012); see also Charras et al. (2014). Participants in their study had to quickly press one of two buttons to classify the sums of visually presented symbolic addition problems as either larger or smaller than a previously presented standard number. Specifically, participants responded to sums of addition problems stated with either two repeated numbers (RN), such as “24 + 24”, or two different numbers (DN), such as “22 + 26”, by classifying them relative to a standard (e.g., 45).

The authors replicated two well-established findings in this set of studies. First, participants were faster for RN than DN problems, indicating the “tie effect” in mental arithmetic (Groen and Parkman, 1972), a cognitive advantage for arithmetic problems with repeated over DN. The tie effect has been attributed to either faster encoding (Blankenberger, 2001) or better memory access (Campbell and Gunter, 2002; Lefevre et al., 2004), thus postulating more efficient processing for repeated over DN.

And secondly, participants of Charras et al. (2012, 2014) were slower and less accurate for sums that were numerically near compared to far from the standard; this outcome reflects a “distance effect” and held for both RN and DN problems. The distance effect with numbers (Moyer and Landauer, 1967) points to the embodied nature of symbol comprehension: instead of performing all number comparisons with equal speed, as a computer does, humans seem to recur to analog magnitude representations which are harder to discriminate when they are more similar (Dehaene et al., 1990; see also Hoedemaker and Gordon, 2014). The “perceptual symbol systems” account of cognition (Barsalou, 1999; Fischer and Coello, 2016) has gained substantial ground across the cognitive sciences generally and focuses on representational instead of processing differences, also for supposedly abstract number concepts which are believed to be represented on an analog “mental number line” (MNL; cf. Fischer and Shaki, 2018).

While neither tie nor distance effect were novel, the interpretation offered by the authors was. Specifically, Charras et al. (2012, 2014) repeatedly found that DN problems generated more errors when their sum was smaller than the standard while RN problems generated more errors when their sum exceeded the standard. The latter observation implies that participants misperceived a sum made from identical operands as less than it was and declared a sum larger than the standard as being smaller, thereby creating an error. The authors likened this latter finding to a perceptual bias for accurately bisected lines, which are typically underestimated in length (Charras and Lupianez, 2009, 2010). In other words, both correctly bisected lines and correctly bisected sums seem to induce an underestimation bias.

Thus, Charras et al. (2012, 2014) offered a novel underestimation account of their finding for tie problems, as follows: In each trial the numbers are initially mapped onto a MNL for comprehension; however, in RN trials this mapping occurs only once because the second number repeats the first. The resulting effort reduction leads to faster response latencies; this is internally monitored and activates “a heuristic linking cognitive effort and magnitude estimation” (Charras et al., 2012, p. 163) which makes smaller than the correct results seem acceptable for RN problems (even though the correct sum actually exceeds the standard), thus creating the asymmetric error distribution. This explanation would effectively constitute a violation of the identity axiom of arithmetic, according to which any sum s = s, regardless of its composition. An example of identity violation has been reported for length productions by Shaki et al. (2015). So is this representational claim correct?

Two arguments seem to contradict the proposal of underestimation of sums computed from ties. First, the idea of tie underestimation contradicts the very characteristic of tie-problems, namely being solved more accurately (as well as faster) than non-tie problems. And secondly, if anything, addition should yield overestimation not underestimation of results, as indicated by the recent literature on operational momentum (OM): Since its discovery (McCrink et al., 2007), several reports with both symbolic and non-symbolic addition tasks showed that normal adult participants, as well as children and infants, tend to overestimate the outcomes of addition problems when either the encoding of operands (in the form of dot clouds) or the production of results (in the form of line lengths or line positions) is somewhat uncertain (for recent review, see Pinheiro-Chagas et al., 2018). Specifically, they either accept larger than the correct numerosities as acceptable outcomes, or produce longer than the correct line lengths. The same overestimation would thus be expected in tie addition problems.

For these reasons we decided to re-asses the underestimation claim of Charras et al. (2012, 2014) for tie addition problems. The evidence provided by Charras et al. (2012, 2014) rests on inferences from latencies and error distributions. Instead, the current study examined the hypothesized underestimation of tie-based results more directly by applying two well-established methods for magnitude estimation in mental arithmetic: the result-to-position pointing task (RPPT) (Experiment 1) and the result-to-line-length production task (RLPT) (Experiment 2). Our findings converge with the authors’ representational claim.

## Experiment 1: Result-To-Position Pointing Task (RPPT)

In the RPPT, participants locate target numbers on a horizontal line where small numbers are located on the left and larger numbers on the right side. This population stereotype is captured in the MNL and leads to systematic biases in many number-related tasks (for reviews, see Fischer and Shaki, 2014, 2018; Winter et al., 2015; Toomarian and Hubbard, 2018). Spatial biases in performance reflect the cognitive operations involved in the retrieval of number concepts, both in children and adults (Siegler and Opfer, 2003; Rouder and Geary, 2014; Bar et al., 2018). The RPPT has been extended from single numbers to the study of mental arithmetic (Pinhas and Fischer, 2008; Pinhas et al., 2014, 2015) where the OM effect was documented as a left-bias in subtraction and a right-bias in addition, supporting the notion of a spatially extended MNL. In the present context, the pointing task clarified whether sums computed from RN addition were indeed underestimated, and would then generate a left-bias, or instead overestimated, as would be reflected in a right-bias.

Another advantage of the RPPT was that, performed on a touch screen, it enabled recording of reaction time (RT, the time interval from stimulus onset to releasing with one’s finger a start location on the touch screen) and movement time (MT, the time from breaking the contact to re-establishing the finger contact with the screen, i.e., from response initiation to response completion). RT is a well-established indicator of response planning and underlying representational complexity (Henry and Rogers, 1960; Fischer, 2001). We would thus expect longer RTs for RN compared to DN trials. MT is sensitive to cognitive effort during response execution (Fitts, 1954; Fischer, 2003). Thus, additional insights about cognitive differences between RN and DN processing would be obtained with our method. A likely candidate process would be the spatial mapping postulated by Charras et al. (2012, 2014): In each trial of their experiment, numbers were initially mapped onto a MNL as part of their comprehension. If that is the case, we can use MT to measure the duration of this mapping process because the mental mapping of numbers onto the MNL and the spatial localization of these same numbers on a physical line are closely related processes (Fischer, 2003; Song and Nakayama, 2008; Dotan and Dehaene, 2013, 2016; Fischer and Hartmann, 2014). According to Charras et al. (2012, 2014) RN trials should have shorter MTs than DN trials.

### Methods

#### Participants

The sample consisted of a convenience sample of 30 female and 2 male (*n* = 32) undergraduate psychology students from the University of Potsdam in Germany, ranging in age from 18 to 30 (mean age = 22.65 years), in exchange for course credit. All participants had normal vision and were native German speakers.

#### Stimuli and Apparatus

The numerical stimuli were taken from the original study by Charras et al. (2012), Experiment 1 and are listed in the Appendix. They were either single numbers (SN, e.g., 26; these served as non-arithmetic baseline) or pairs of numbers with a plus operator, constituting an addition problem. The additions were manipulated as either a RN addition (e.g., 13 + 13) or a DN addition (11 + 15) while controlling result size. DN addition resulted from RN addition by subtracting n-2 from the first operand and adding n + 2 to the second operand, thus creating a constant split of 4 between operands. There were 25 stimuli in each of the three stimulus categories and results varied from 6 to 96 (see Appendix).

Stimuli were presented on a 55-inch Iiyama touch screen in landscape orientation that was tilted about 45 degrees away from the participant and with its lower edge approximately at the belt height of the standing participant. The screen resolution was 1920 × 1080 pixels. The experiment was designed and controlled using the open source Python library Expyriment© (Krause and Lindemann, 2014).

#### Design

Each stimulus was presented 4 times, resulting in 300 trials in total. These trials were presented in a new randomized sequence to each participant.

#### Procedure

The experiment was completed in a quiet and dimly lit room with only the experimenter present. Before commencing with the task, participants signed an informed consent form. Participants were advised to stand comfortably in front of the screen center as the experiment lasted approximately 40 min. Participants read the instructions on the screen before starting the experiment. Participants were asked to use the right forefinger during the experiment to indicate their answers.

**FIGURE 1:**
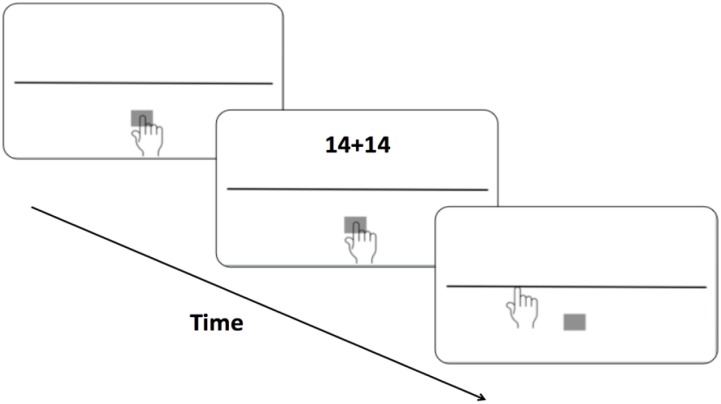
Illustration of event sequence in an RN trial of the RPPT (not to scale).

A typical trial is shown in Figure 1. On the screen participants saw a 1500 pixels long horizontal black line (three pixels wide) 90 pixels below the vertical screen center with a start box (gray square, 50 pixels length) at a distance of 290 pixels below its midpoint. This line represented the magnitude 100, thus each number occupied 15 pixels on the continuum. No anchors or flankers were provided to avoid perceptual distortions (Fischer, 1994). In each trial the participant had to touch the start box until the stimulus digit(s) appeared 135 pixels above the center of the screen (225 pixels above the midpoint of the line). Stimulus onsets were randomly delayed from 250 to 750 ms from registration of the start box contact to prevent anticipatory responses. Each stimulus was shown until the participant had released and then touched the screen again; these landing coordinates were registered. A blank screen followed for 1000 ms and the next trial started when the start box was touched again.

### Results

RT was defined as the time from onset of the numerical stimulus to the release of the start button. MT was the time from releasing the start button to touching the screen again. We also computed horizontal and vertical landing errors as follows: the center of the 15-point continuum representing each number was regarded as the exact position of the respective number. For example, the horizontal pixel value of 83 represented the correct location of target number 6, which extended from 76 to 90 pixels. Deviation along the x-axis was then computed by subtracting the correct result from the registered landing coordinate. Vertical deviation was computed by subtracting the y-position of the line from the recorded landing position.

Three participants were excluded from the data analysis because of an incomplete data set due to equipment malfunction, leaving data from 29 participants for analysis. All trials with RT below 100 ms or above 3000 ms, with MT below 200 ms or above 5000 ms, or an answer that deviated on the y-axis by more than 50 pixels from the response line were removed as non-representative behavior (2.9% of trials). In a subsequent trimming procedure, all data points which deviated by more than two standard deviations from an individual’s mean in RT, MT, horizontal or vertical deviation were successively removed from the data (13.7% of trials).

#### Horizontal Deviations

**FIGURE 2:**
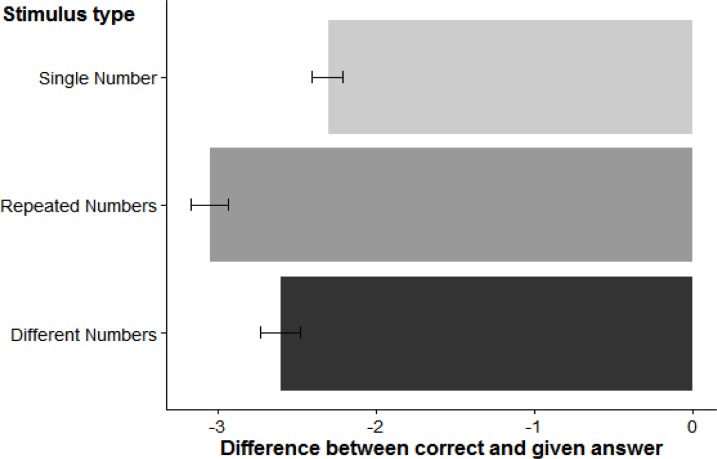
Horizontal deviations (in pixels) for the three stimulus categories. Error bars represent one standard error of the mean.

An analysis of variance (ANOVA) on the trimmed data evaluated the effect of stimulus category (3 levels: RN, Different Number, Single Number) on horizontal deviations. The results are visualized in Figure 2. We found a reliable effect of stimulus category, *F*(2, 56) = 9.59, *η^2^_p_* = 0.20, *p* < 0.001; *t*-tests showed that all landing positions were significantly to the left of the true target location (all *p* < 0.001) and that there was a reliable difference between landing coordinates for DN compared to RN trials, *t*(28) = 2.84, *p* < 0.01. Moreover, SN trials showed reliably smaller left bias than RN trials, *t*(28) = 4.56, *p* < 0.001, but not when compared to DN trials, *t*(28) = 1.49, *p* > 0.05.

#### Reaction Times

**FIGURE 3:**
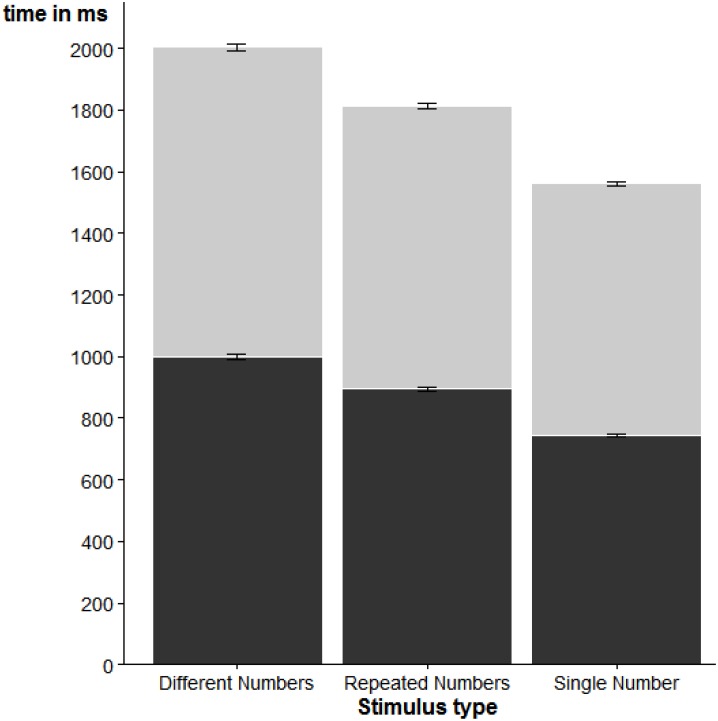
Average RT (dark portion) and MT (light portion) for the different stimulus categories. Error bars represent one standard error of the mean.

A second ANOVA evaluated the effect of stimulus category on RT. We found again a reliable effect of stimulus category, *F*(2, 56) = 19.70, *η^2^_p_* = 0.41, *p* < 0.001, indicating that SN trials led to faster response initiation than RN trials, which in turn led to faster response initiation than DN trials. *Post-hoc* *t*-tests showed that all pairwise contrasts were statistically reliable, *p* < 0.001. These results are visualized in Figure 3.

#### Movement Times

A final ANOVA evaluated the effect of stimulus category on MT. The results are also shown in Figure 3. We found again a reliable effect of stimulus category, *F*(2, 56) = 30.27, *η^2^_p_* = 0.52, *p* < 0.001, due to fastest movement completion for SN trials, followed by RN trials, and slowest movement completions for DN trials. As with RT, the pairwise *t*-tests showed that all contrasts were statistically reliable, all *p* < 0.01.

The regression of MT on RT revealed no reliable effect, *p* > 0.05.

### Discussion

In Experiment 1, we adopted the RPPT and found support for the proposal made by Charras et al. (2012, 2014) that RN problems activate cognitively smaller result magnitudes than DN problems with controlled result sizes. Their evidence from response biases was supplemented here by evidence from spatial biases in a pointing task in which smaller numbers are associated with left space.

Further information in support of the underestimation hypothesis was obtained from the chronometric results. They confirm not only the differential ease of processing for RN compared to DN problems (a finding that is well-established, see Introduction), but also provide, for the first time, a direct measurement of the differential duration of the hypothetical mapping process. By interpreting MT as an indicator for spatial mapping processes, we found that, consistent with the claim of Charras et al. (2012, 2014), the mapping of numbers onto a MNL was faster (and thus easier) for RN compared to DN problems. The fact that RT and MT were negatively correlated is not problematic because it occurred in all conditions equally. Instead, this observation actually validates our argument that the crucial mapping process, stipulated by Charras et al. (2012, 2014), can indeed be captured by measuring physical movement times. We will elaborate on this argument in the General Discussion.

The present results replicate an underestimation bias in addition, thus supporting the notion that we systematically violate the identity axiom of formal arithmetic. We note that the sample consisted largely of female participants and there might be a problem with generalizing these findings although we are unaware of gender biases in this pointing task. However, a more pertinent problem with the present results is the spatial nature of the pointing task which might contaminate the assessment of the cognitive representation of numbers by introducing space-related associations that do not normally occur when thinking about numbers (for full discussion, see Fischer and Shaki, 2018). Arguably, the underestimation effect found by Charras et al. (2012, 2014) was equally contaminated by extraneous spatial associations because they used two lateral response buttons. A task without spatially biased features would remove the possible contribution of the sign-space association to the present results and clarify the sole effect of anchoring bias. This would in turn add further weight to the conclusion that sums computed in RN problems are underestimated. Finally, the evidence for underestimation of RN so far remains indirect because it required an inference from either biased choices (Charras et al., 2012, 2014) or from temporal-spatial performance patterns (Experiment 1 above) on the underlying quantity representation. For these reasons, we adopted a non-spatial task in Experiment 2 to provide more direct evidence of underestimation while also removing (or at least reducing) any spatial contamination from the assessment. Our choice of task was inspired by well-established psychophysical procedures for assessing perception with so-called production tasks. In these tasks participants generate their own responses and results do not need to be adapted or calibrated to the specific observer because idiosyncratic aspects of responding affect all values provided by any given participant (cf. Gescheider, 1997).

## Experiment 2: Result-To-Line-Length Production Task (RLPT)

Several reasons suggested the use of RLPT in order to provide further converging evidence for an underestimation of RN. First, the RLPT allows direct assessment of the quantity associated with a specific numerical concept (Shaki et al., 2015, 2018): Participants equate the length of a horizontal line so that it corresponds to their internal magnitude for the result of an arithmetic computation. Thus, the resulting length is a direct indicator of the cognitive magnitude. Adopting this rationale, we expected to see shorter lines for RN compared to DN problems.

Another benefit of using the specific RLPT we describe below is to (largely) remove spatial biases from the task. Note that participants produced self-calculated arithmetic results by changing bi-directionally the length of a horizontally extended line, using vertically aligned keys. These features ensured that participants evaluated magnitude representations of outcomes without response-related directional spatial biases (cf. Fischer and Shaki, 2016).

### Methods

#### Participants

Eighteen students (mean age 23.2, SD = 3.69 years, 5 male, 2 left-handed) from Ariel University participated for course credit. They all provided written informed consent prior to the beginning of the experiment, using a protocol approved by the Ariel University Institutional Review Board.

#### Stimuli and Apparatus

For reasons of efficiency, the stimuli set consisted a subset of the stimuli we used in Experiment 1. Nine outcomes of 14, 26, 38, 44, 50, 56, 62, 74, and 86 were presented as SN (baseline) or manipulated as either RN addition or DN addition (in the same way as in Experiment 1). Presentation of instructions, stimuli, timing and response recording were controlled by in-house-software. Stimuli (30 points bold Times New Roman, black on white) were presented on a 19-inch (1280 × 1024 pixels) display. The horizontal lines were three pixels (0.75 mm) tall. Responses were made with up- and down-arrow-keys of a standard QWERTY keyboard.

#### Design

**FIGURE 4:**
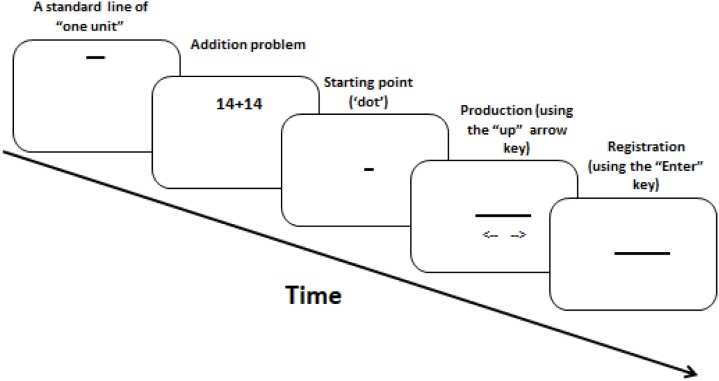
Illustration of the RLPT method used in Experiment 2. Directional arrows in the “Production” panel illustrate bilateral line extension and were not actually shown.

Each trial started with a horizontal line of “one unit” (100 pixels) presented at the display center. Based on this standard, participants produced the line length corresponding to the magnitude of a result (calculation task) or a single number (baseline). Pressing an arrow-key initiated the presentation of a “dot” (2 pixels); each additional press adjusted the line-length bi-directionally by 2 pixels (up/down arrow for longer/shorter lines, respectively). Continuous pressing adjusted line length at a rate of 30 Hz. Figure 4 illustrates the trial sequence. Each arithmetic problem or single number appeared 4 times (9 outcomes × 2 problem types 4 repetitions = 72 trials; 9 single numbers × 4 repetitions = 36 baseline trials), yielding 108 trials per participant.

#### Procedure

Participants sat approximately 50 cm from the display. Response keys were centered under the display. Each trial started with the standard line of “one unit” for 400 ms, followed after 200 ms blank screen by the stimulus for 600 ms, followed by another blank screen until participants pressed an arrow key to trigger the starting point display of the ’dot. Both increasing and decreasing line adjustments were permitted until participants pressed the “Enter” key to register their response. This was followed by the next trial without feedback. A practice block preceded the experiment.

### Results

**FIGURE 5:**
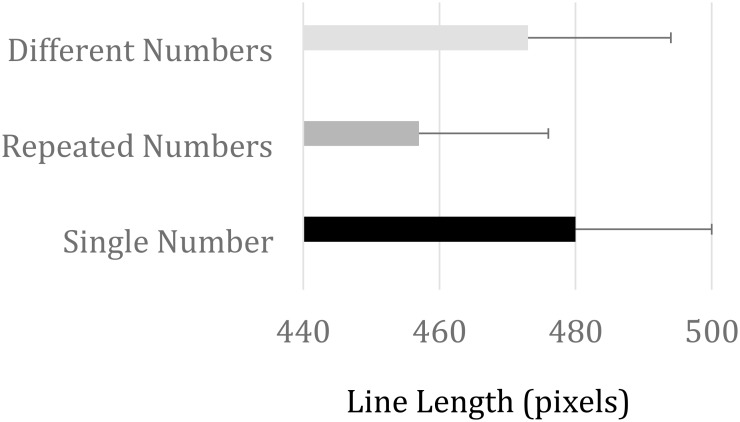
Horizontal line length productions (in pixels) for the three stimulus categories. Error bars represent one standard error of the mean.

An analysis of variance (ANOVA) evaluated the effect of Outcome magnitude (9 possible outcomes: 14, 26, 38, 44, 50, 56, 62, 74, and 86) and stimulus condition (3 levels: RN, DN, and SN) on line length production. A main effect of outcome magnitude, *F*(8, 136) = 222.53, *MSE* = 7,750, *p* < 0.001, *η^2^_p_* = 0.93, confirmed task compliance. Average line lengths for outcomes 14, 26, 38, 44, 50, 56, 62, 74, and 86 were 168, 272, 378, 422, 529, 519, 564, 644, and 733 pixels, respectively. More interesting, we found a reliable main effect of stimulus condition, *F*(2, 34) = 7.70, *MSE* = 2,980, *p* < 0.01, *η^2^_p_* = 0.31, revealed shorter line length for RN compared to DN and SN (457, 473, and 480 pixels, respectively). This is depicted in Figure 5. Paired *t*-tests between conditions showed that participants produced significantly shorter lines in RN compared to DN [*t*(17) = 2.89, *p* < 0.01] and SN [*t*(17) = 4.06, *p* < 0.001] conditions, while no reliable difference was found between DN and SN conditions [*t*(17) = 1.12, *p* = 0.28].

### Discussion

Our second experiment adopted a RLPT and replicated the findings of Experiment 1 that sums computed from RN lead to significant underestimation of the underlying result magnitude. This outcome provided direct support for the proposal of Charras et al. (2012, 2014). Our finding was obtained with a method that has previously been argued to reduce or remove spatial biases from the assessment itself, thus making it less likely that the outcome reflects extraneous factors. On the other hand, this method requires a somewhat artificial behavior which might raise concerns about the generalizability of results to real-life arithmetic. With this in mind we can now turn to a general discussion of our findings and their implications for our understanding of mental arithmetic.

## General Discussion

This study was motivated by the report of an apparent anomaly in mental arithmetic. Specifically, Charras et al. (2012, 2014) inferred from the error pattern in their data that results of RN addition problems (so-called “tie problems”) were underestimated relative to results of DN problems. This interpretation stands in conflict with the established superior performance on tie compared to non-tie problems, and with a substantial literature reporting an OM effect according to which results of addition problems are normally overestimated (and results of subtraction problems underestimated).

Here we applied a pointing task and a line production task to clarify that additions are indeed underestimated and that RN results are reliably more underestimated compared to DN results: In Experiment 1, 29 adult participants localized RN results significantly further left on a visually presented number line than DN results. This spontaneous left-bias is an established signature of underestimation (Siegler and Opfer, 2003; Pinhas and Fischer, 2008; for review see Fischer and Shaki, 2014) and confirms the interpretation of Charras et al. (2012, 2014) that RN results are underestimated. The fact that all responses were, on average, to the left of the correct location is not unexpected given the normal tendency to bisect lines to the left of their true center (cf. Jewell and McCourt, 2000). The fact that even DN problems did not induce OM is particularly remarkable because it shows that task details can strongly affect the nature of the observed bias. We attribute this aspect of our results to the fact that we used an ascending order of operands throughout, thus inducing strong anchoring on the smaller first operand values. This *post-hoc* account can be tested in a new study.

Our pointing task also included measurements of RT as indicator of response planning and MT as indicator of the spatial mapping of numbers to assess the relative difficulty of RN and DN conditions. We found that RN results were significantly easier to compute than DN results, as evidenced by reliably faster response planning. These results replicate the established processing advantage of tie problems and also the observations of Charras et al. (2012, 2014). Moreover, our MT results provide support for their second claim, namely that the subsequent spatial mapping is easier for RN compared to DN problems. The cognitive mapping of numbers onto the MNL and the spatial mapping of a number onto a physical number line are closely related processes, as evidenced by a series of number-related movement tasks (Fischer, 2003; Song and Nakayama, 2008; Dotan and Dehaene, 2013, 2016; Fischer and Hartmann, 2014). The fact that participants were able to flexibly complete some of the necessary cognitive steps during either RT or MT, as established by their negative correlation, supports our claim that this mapping process can be captured by measuring MTs.

In Experiment 2, line length productions were adopted as performance measure to provide more direct evidence for the hypothesized underestimation of results when computed from RN rather than DN operands. Again consistent with the prediction of Charras et al. (2012, 2014), 18 healthy adult participants produced shorter line lengths in the RN compared to the DN and SN tasks. Given that the task was performed with response keys that were orthogonal to the relevant (horizontal) stimulus dimension, and the stimulus itself (the line) was modified bi-laterally and bi-directionally, we are confident that magnitude assessments were not contaminated by prevalent spatial-numerical biases (cf. Shaki and Fischer, 2018).

The current data suggest a re-evaluation of the unanimous processing advantage of tie problems because this apparent advantage comes with a representational bias. Regardless of whether the tie advantage is due to faster encoding (Blankenberger, 2001) or better memory access (Campbell and Gunter, 2002; Lefevre et al., 2004), once the task introduces uncertainty regarding the expression of results, a tendency to violate the identity axiom of arithmetic by underestimating tie outcomes becomes evident. While there may be no immediate practical implications of this discovery, this fact does have theoretical implications.

This finding supports the arithmetic heuristics and biases (AHAB) model of cognitive biases in mental arithmetic, according to which three sources of error can contaminate mental arithmetic outcomes (Shaki et al., 2018): First, the use of a heuristic according to which we accept “more” during additions and “less” during subtractions; this OM component predicts overestimation of addition outcomes (McCrink et al., 2007). Second, an association between operators and space which relates right space to addition and to larger magnitudes (Pinhas et al., 2014; Mathieu et al., 2017); this OM component is only effective in tasks with spatial aspects, such as pointing to results of additions on a number line (RPPT; e.g., Pinhas and Fischer, 2008; Pinhas et al., 2014, 2015). And finally, an anchoring effect from the first number (Tversky and Kahneman, 1974); importantly, this OM component can explain reverse OM, e.g., when subtraction problems have larger first operands than addition problems due to controlled result size between operations. In the studies conducted by Charras et al. (2012, 2014) this was the case.

However, while reverse OM – and thus underestimation of addition outcomes - is predicted by AHAB as a result of anchoring on small first operands, the ordering of means in the present study is not. Note that our DN problems all had smaller first than second operands compared to RN problems, yet they induced less underestimation compared to RN problems. This unexpected lack of stronger anchoring on small numbers with smaller first operands suggests that operand order is a further factor that must be taken into consideration when predicting OM. Specifically, we suggest that ascending operand order (which we adopted for all DN problems) is congruent with the mental number line, thus inducing a bias similar to the processing fluency heuristic postulated by Charras et al., which in turn leads to larger perceived results in DN compared to RN problems. Further work is needed to test this proposed extension of AHAB and the relative contribution of biases when compared to SN. One particularly diagnostic test would be to manipulate operand order and compare results in subtraction tasks; ideally this could be done in separate blocks to see if there is a general operation-specific mental set that affects (and eliminates) the heuristic component of OM.

In summary, the present study supports the view that tie problems have not only a processing advantage in terms of fast and accurate problem solving but also a processing penalty in the form of a distorted magnitude representation. This representational distortion violates a fundamental axiom of arithmetic and constitutes a novel bias that must be considered when understanding errors in simple mental arithmetic.

## Ethics Statement

The first experiment was conducted at Potsdam University and complied with ethical guidelines of the British Psychological Society (BPS). The second experiment was carried out in accordance with the recommendations of “Ariel University institutional review board” with written informed consent from all subjects. All subjects gave written informed consent in accordance with the Declaration of Helsinki.

## Author Contributions

All authors listed have made a substantial, direct and intellectual contribution to the work, and approved it for publication.

## Conflict of Interest Statement

The authors declare that the research was conducted in the absence of any commercial or financial relationships that could be construed as a potential conflict of interest.

## Appendix

**Table 1:** Stimuli used in Experiment 1. Tie problems appear in the Repeated Numbers (RN) column.

SN	RN	DN
6	3 + 3	1 + 5
8	4 + 4	2 + 6
12	6 + 6	4 + 8
14	7 + 7	5 + 9
18	9 + 9	7 + 11
20	10 + 10	8 + 12
26	13 + 13	11 + 15
30	15 + 15	13 + 17
32	16 + 16	14 + 18
38	19 + 19	17 + 21
42	21 + 21	19 + 23
44	22 + 22	20 + 24
50	25 + 25	23 + 27
54	27 + 27	25 + 29
56	28 + 28	26 + 30
62	31 + 31	29 + 33
66	33 + 33	31 + 35
68	34 + 34	32 + 36
74	37 + 37	35 + 39
78	39 + 39	37 + 41
80	40 + 40	38 + 42
86	43 + 43	41 + 45
90	45 + 45	43 + 47
92	46 + 46	44 + 48
96	48 + 48	46 + 50

*SN, Single Number; RN, Repeated Number Addition; DN, Different Number Addition.*
